# A double-feature mitochondrial proteome exploration show

**DOI:** 10.1093/plphys/kiae056

**Published:** 2024-02-07

**Authors:** Sebastián R Moreno, José Manuel Ugalde

**Affiliations:** Assistant Features Editor, Plant Physiology, American Society of Plant Biologists; Sainsbury Laboratory, University of Cambridge, Bateman Street, Cambridge CB2 1LR, UK; Assistant Features Editor, Plant Physiology, American Society of Plant Biologists; Institute of Crop Science and Resource Conservation (INRES)-Chemical Signalling, University of Bonn, Friedrich-Ebert-Allee 144, 53113 Bonn, Germany

In flowering plants, RNA editing is crucial in post-transcriptional RNA maturation. RNA editing involves C-to-U deaminations, affecting about 500 editing sites in mitochondria and 40 editing sites in plastids (Takenaka et al. 2013). RNA editing is essential for restoring functional proteins and can potentially influence protein activity, localization, or protein–protein interaction (Small et al. 2023). Remarkably, the lack of detected proteins corresponding to non-edited or partially edited transcripts suggests that RNA editing acts primarily as a repair mechanism rather than providing a regulatory advantage in plants.

Proteomic approaches, particularly mass spectrometry (MS)-based ones, have facilitated the identification of proteins in plant organelles (Nelson et al. 2013, Mueller et al. 2014; Salvato et al. 2014; Senkler et al. 2017; Fuchs et al. 2020). Several quantitative proteomics studies of plant mitochondria and chloroplasts are integrated into platforms such as the Arabidopsis PeptideAtlas and Proteomexchange platform (Vizcaíno et al. 2014; van Wijk et al. 2021). However, available datasets have not considered the abundance of non-edited transcripts, which still makes the relevance of non-edited transcripts in the plant organellar proteomic landscape uncertain. In a previous *Plant Physiology* issue, van Wijk et al. (2024) reanalyzed the Arabidopsis PeptideAtlas project, focusing on RNA editing. They identified 167 predicted edit sites and found that major frequency sites (>50%) predominantly accumulate in edited form at the protein level, suggesting that RNA editing is required for stable protein accumulation. However, a comprehensive mitochondrial proteome of *Arabidopsis* with considerable depth is still missing, leaving the question of whether undetected non-edited proteins are functional unanswered.

In this issue of *Plant Physiology*, Rugen et al. (2024) address this gap in knowledge by performing a new shotgun proteomic strategy. The authors isolated mitochondria from a non-green cell culture and performed a protein isolation. Although trypsin digestion sufficed for conventional shotgun sequencing, the researchers opted for five additional proteases to ensure comprehensive proteome coverage (Fig. 1A).

**Figure 1.**
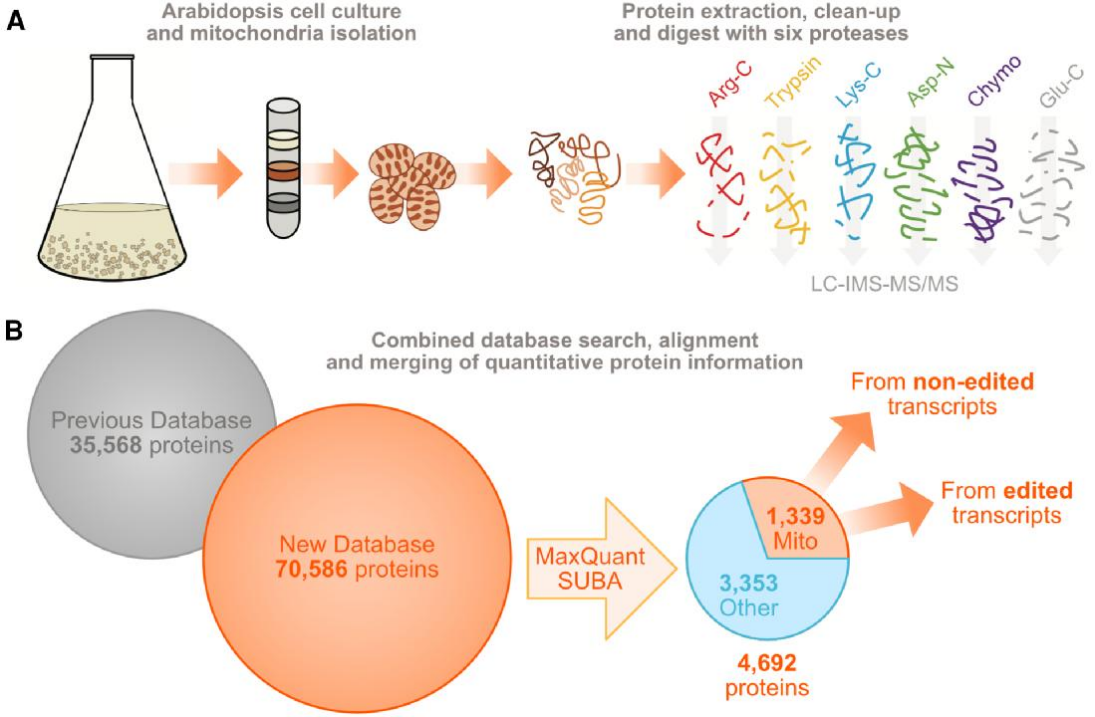
Experimental pipeline. **A)** A mitochondrial fraction was isolated from a liquid non-green cell culture. Proteins were extracted from this fraction and digested with six different proteases, resulting in smaller peptides that LC-IMS-MS/MS analyzed. **B)** The results were combined and processed in MaxQuant, leading to an updated *Arabidopsis* mitochondrial protein database, including, for the first time, proteins from edited and non-edited transcripts. Figure adapted from Rugen et al. (2024) by J.M.U using Affinity Designer version 2.3.1.

For data evaluation, an extended Arabidopsis protein database was used, which was combined from TAIR10 and Araport11 and includes 70,586 protein entries (Fig. 1B). To extend the database as much as possible, the team combined the latest annotated databases TAIR10 and Araport11. In addition, to dig deeper into the enigmatic role of potentially non-edited proteins, the authors incorporated permutated amino acid sequences, including all potential RNA editing sites. Employing LC, IMS, and MS/MS, a comprehensive and updated platform for exploring the mitochondrial proteome was developed, leading to the identification of 4,692 proteins, surpassing the coverage achieved in previous studies (Fuchs et al. 2020). Applying MaxQuant and SUBA for data filtration, the initially identified 4,692 proteins were narrowed down to 1,339 predicted to reside in mitochondria, achieving 81% purity in the final mitochondrial proteome. Notably, the groundbreaking discovery emerged that proteins originating from non-edited transcripts can integrate into native ribosomes and ATP synthase complexes (Fig. 1B).

Additionally, the researchers successfully detected proteins originating from non-edited RNAs, leveraging their new database to differentiate between edited and non-edited proteins. This innovative approach allows for the identification and quantification of specific protein variants that create a more heterogeneous protein landscape, exemplified by the detection of partially edited versions of RPS3 and ATP4. Further analysis could solve the biological implications of the protein heterogeneity detected in these proteins.

In summary, this study not only fills a significant knowledge gap but also introduces a robust methodology for exploring and understanding the intricacies of the plant mitochondrial proteome. Despite a few exceptions, the authors observed that most proteins are produced from fully edited transcripts, regardless of the editing rates at the individual sites. As a result, this study represents an innovative discovery that paves the way for further exploration of proteins from partially edited transcripts. For instance, further exploration of the role of RPS3 and ATP4 could help us to understand whether these proteins are a consequence of inadequate quality control or if organelle protein heterogeneity contributes to an additional regulatory step within flowering plants.

## Dive Curated Terms

The following phenotypic, genotypic, and functional terms are of significance to the work described in this paper:

ATP CHEBI: CHEBI:15422

ATP4 Gramene: ATMG00640
ATP4 Araport: ATMG00640
